# Supplementary material for: Genome-wide RNA polymerase stalling shapes the transcriptome during aging
Source: Nat Genet. 2023 Jan 19;55(2):268–79. doi: 10.1038/s41588-022-01279-6 (PMC9925383; doi:10.1038/s41588-022-01279-6)
Supplement: Supplementary file 1 — Reporting Summary [file 41588_2022_1279_MOESM1_ESM.pdf]

nature portfolio

Corresponding author(s): Joris Pothof

Last updated by author(s): Sep 9, 2022

Reporting Summary

Nature Portfolio wishes to improve the reproducibility of the work that we publish. This form provides structure for consistency and transparency in reporting. For further information on Nature Portfolio policies, see our Editorial Policies and the Editorial Policy Checklist.

Statistics

For all statistical analyses, confirm that the following items are present in the figure legend, table legend, main text, or Methods section.

n/a	Confirmed	
☐	☒	The exact sample size (*n*) for each experimental group/condition, given as a discrete number and unit of measurement
☐	☒	A statement on whether measurements were taken from distinct samples or whether the same sample was measured repeatedly
☐	☒	The statistical test(s) used AND whether they are one- or two-sided<br>*Only common tests should be described solely by name; describe more complex techniques in the Methods section.*
☒	☐	A description of all covariates tested
☐	☒	A description of any assumptions or corrections, such as tests of normality and adjustment for multiple comparisons
☐	☒	A full description of the statistical parameters including central tendency (e.g. means) or other basic estimates (e.g. regression coefficient) AND variation (e.g. standard deviation) or associated estimates of uncertainty (e.g. confidence intervals)
☐	☒	For null hypothesis testing, the test statistic (e.g. *F*, *t*, *r*) with confidence intervals, effect sizes, degrees of freedom and *P* value noted<br>*Give P values as exact values whenever suitable.*
☒	☐	For Bayesian analysis, information on the choice of priors and Markov chain Monte Carlo settings
☒	☐	For hierarchical and complex designs, identification of the appropriate level for tests and full reporting of outcomes
☒	☐	Estimates of effect sizes (e.g. Cohen's *d*, Pearson's *r*), indicating how they were calculated

Our web collection on statistics for biologists contains articles on many of the points above.

Software and code

Policy information about availability of computer code

Data collection	No software was used for data collection
Data analysis	Sequence-read quality control was performed by using Fastqc (v0.11.3; http://www.bioinformatics.babraham.ac.uk/projects/fastqc/)<br>Sequencing reads were pre-processed with Trimmomatic (v0.35) [PMID: 24695404]<br>RNA-seq reads were aligned by y using Tophat2 (v2.0.9) [PMID: 19289445].<br>Htseq (v0.6.0) [PMID: 25260700] was used to quantify reads<br>Bowtie2 [PMID: 22388286] was used to align ChIP-seq reads.<br>FastQScreen (v0.14.0). https://www.bioinformatics.babraham.ac.uk/projects/fastq_screen/<br>FastQC (v0.11.9). https://www.bioinformatics.babraham.ac.uk/projects/fastqc/<br>K-means clustering software (Cluster 3.0; http://bonsai.hgc.jp/~mdehoon/software/cluster/software.htm)<br>HOMER v4.11 software for ChIP-seq profile generation [PMID: 20513432]<br>Nucleotide composition was determined by Qualimap (v2.21) [PMID: 26428292].<br>R project for statistical computing. https://www.r-project.org/<br>EdgeR (DOI: 10.18129/B9.bioc.edgeR)<br>BioConductor seqTools (Kaisers W (2013). R package version 1.2.0.<br>IRanges packages (2.30.1) [PMID: 23950696].<br>Analysis of EU-seq read abundance at splicing donor and acceptor sites was carried out by a custom-written script:<br>Splicingdonor&acceptorfinder.py<br>Binning genes was carried out by a custom-updated script from the existing code from RSeQC (v3.0.1) geneBody_coverage.py:<br>K_binning.py<br>Ingenuity Pathway Analysis, QIAGEN.<br>Gene set enrichment analysis (GSEA). https://www.gsea-msigdb.org/gsea/index.jsp<br>Kyoto Encyclopedia of Genes and Genomes (KEGG). https://www.genome.jp/kegg/pathway.html

Fiji (Image J 1.53q) https://fiji.sc/

Alternative splicing events were detected by Astalavista (v4.0) [PMID: 25577392]
Samtools (v1.9) [PMID: 19505943]
Bedtools (v2.27.1) [PMID: 20110278].
GC content and DNA methylation status were detected by Qualimap (v2.21) and deepTools (v2.0) [PMID: 27079975].
Graphpad Prism, Version 7.05. (WWW.graphpad.com)

For manuscripts utilizing custom algorithms or software that are central to the research but not yet described in published literature, software must be made available to editors and reviewers. We strongly encourage code deposition in a community repository (e.g. GitHub). See the Nature Portfolio guidelines for submitting code & software for further information.

Data

Policy information about availability of data

All manuscripts must include a data availability statement. This statement should provide the following information, where applicable:

- Accession codes, unique identifiers, or web links for publicly available datasets
- A description of any restrictions on data availability
- For clinical datasets or third party data, please ensure that the statement adheres to our policy

The nascent RNA-seq and ChIP-seq data discussed in this publication have been deposited in NCBI's Gene Expression Omnibus, and are accessible through: https://www.ncbi.nlm.nih.gov/sra/PRJNA603447
The following publicly available data was used:
Mouse ribosomal DNA (BK000964.3) from NCBI.
Mouse mitochondrial sequences (UCSC, mm10) from UCSC genome browser.
Mouse reference genome (GRCm38/mm10) from Ensembl.
Human reference genome (hg19).
C.elegans reference genome (ce10).
Published datasets were downloaded from European Nucleotide Archive.
Total RNA-seq data from human tendon [PMID:25888722].
Total RNA-seq data from Caenorhabditis elegans [PMID: 29298683]
DNA methylation (PRJNA376757) [PMID: 28249716],
Histone H3K27ac and H3K4me3 data (PRJNA281127) [PMID: 30858345]
MNase-seq (GSE58005) [PMID: 25437555]
Reactome Pathway Databases, Version 79
Aging Perturbations from GEO down datasets from Enrichr
Kegg database, Release 99.0
QIAGEN Ingenuity Pathway Analysis

Field-specific reporting

Please select the one below that is the best fit for your research. If you are not sure, read the appropriate sections before making your selection.

☒ Life sciences ☐ Behavioural & social sciences ☐ Ecological, evolutionary & environmental sciences

For a reference copy of the document with all sections, see nature.com/documents/nr-reporting-summary-flat.pdf

Life sciences study design

All studies must disclose on these points even when the disclosure is negative.

Sample size	Sample sizes were n=3 mice / group for all experiments.
Data exclusions	No data was excluded.
Replication	Experiments were performed at minimum of 3 times. All replicates were successful.
Randomization	Our study design did not require randomization.
Blinding	Our study design did not require blinding

Reporting for specific materials, systems and methods

We require information from authors about some types of materials, experimental systems and methods used in many studies. Here, indicate whether each material, system or method listed is relevant to your study. If you are not sure if a list item applies to your research, read the appropriate section before selecting a response.

Materials & experimental systems

n/a	Involved in the study
☐	☒ Antibodies
☐	☒ Eukaryotic cell lines
☒	☐ Palaeontology and archaeology
☐	☒ Animals and other organisms
☒	☐ Human research participants
☒	☐ Clinical data
☒	☐ Dual use research of concern

Methods

n/a	Involved in the study
☐	☒ ChIP-seq
☒	☐ Flow cytometry
☒	☐ MRI-based neuroimaging

Antibodies

Antibodies used

RNAPII-ser2p (Abcam, ab5095)
RNAPII-ser5p (Abcam, ab5131)
(Total) RNAPII RPB1-NTD (Cell signaling, D8L4Y)
Alexa-fluor-594-RPB1 (Biolegend, 664908)
Phospho-ATM (Ser1981, Cell Signaling #4526)
Phospho-Histone H2A.X (Ser139, Cell Signaling; #9718)

Validation

Validation for all antibodies is available at the manufacture's websites:
RNAPII RPB1-NTD (Cell signaling, D8L4Y) : https://www.cellsignal.de/products/primary-antibodies/rpb1-ntd-d8l4y-rabbit-mab/14958
RNAPII-ser2p (Abcam, ab5095) : https://www.abcam.com/rna-polymerase-ii-ctd-repeat-ysptsps-phospho-s2-antibody-ab5095.html
RNAPII-ser5p (Abcam, ab5131) : https://www.abcam.com/rna-polymerase-ii-ctd-repeat-ysptsps-phospho-s5-antibody-ab5131.html
Alexa-fluor-594-RPB1 (Biolegend, 664908) : https://www.biolegend.com/de-de/products/alexa-fluor-594-anti-rna-polymerase-ii-rpb1-antibody-12144
Phospho-ATM (Ser1981, Cell Signaling #4526) : https://www.cellsignal.de/products/primary-antibodies/phospho-atm-ser1981-10h11-e12-mouse-mab/4526
P-H2A.X (Ser139, Cell Signaling; #9718) : https://www.cellsignal.de/products/primary-antibodies/phospho-histone-h2a-x-ser139-20e3-rabbit-mab/9718

Eukaryotic cell lines

Policy information about cell lines

Cell line source(s): mouse dermal fibroblasts isolated in the Erasmus MC experimental animal facility

Authentication: None of the cell lines used were authenticated

Mycoplasma contamination: Cell lines were confirmed mycoplasma free.

Commonly misidentified lines (See ICLAC register): *Name any commonly misidentified cell lines used in the study and provide a rationale for their use.*

Animals and other organisms

Policy information about studies involving animals; ARRIVE guidelines recommended for reporting animal research

Laboratory animals

15 and 104 weeks old male wild type mice in a F1 C57BL6J/FVB (1:1) hybrid background.
DNA repair deficient premature ageing mouse models in F1 C57BL6J/FVB (1:1) hybrid background:
4 and 10 weeks old male Ercc1Δ/– mutants [PMID: 9197240].
7 and 14 weeks old male Xpg-/- mutants [PMID: 25299392].

Wild animals: No wild animals were used in the study.

Field-collected samples: No field collected samples were used in the study.

Ethics oversight: Animal experimentation followed the “Animal Welfare Act” of the Dutch government, named: the “Guide for the Care and Use of Laboratory Animals” as standard. License number: GGO 97-187, protocol 139-12-18, EMC number: 2767

Note that full information on the approval of the study protocol must also be provided in the manuscript.

ChIP-seq

Data deposition

☒ Confirm that both raw and final processed data have been deposited in a public database such as GEO.

☐ Confirm that you have deposited or provided access to graph files (e.g. BED files) for the called peaks.

Data access links<br>*May remain private before publication.*	sra: PRJNA603447
Files in database submission	RNA Polymerase ChIP-seq from liver (triplicates):<br>Total RNAPII ChIP-seq adult 1<br>Total RNAPII ChIP-seq adult 2<br>Total RNAPII ChIP-seq adult 3<br>Total RNAPII ChIP-seq old 1<br>Total RNAPII ChIP-seq old 2<br>Total RNAPII ChIP-seq old 3<br>RNAPII-ser2 ChIP-seq adult 1<br>RNAPII-ser2 ChIP-seq adult 2<br>RNAPII-ser2 ChIP-seq adult 3<br>RNAPII-ser2 ChIP-seq old 1<br>RNAPII-ser2 ChIP-seq old 2<br>RNAPII-ser2 ChIP-seq old 3<br>RNAPII-ser5 ChIP-seq adult 1<br>RNAPII-ser5 ChIP-seq adult 2<br>RNAPII-ser5 ChIP-seq adult 3<br>RNAPII-ser5 ChIP-seq old 1<br>RNAPII-ser5 ChIP-seq old 2<br>RNAPII-ser5 ChIP-seq old 3<br>Input ChIP-seq adult<br>Input ChIP-seq old<br><br>Nascent RNA-seq (EU-seq) from UV-irradiated cells:<br>EU-seq in vitro control (no UV)<br>EU-seq in vitro 2J/m2 UVC<br>EU-seq in vitro 4J/m2 UVC<br>EU-seq in vitro 6J/m2 UVC<br><br>Nascent RNA-seq (EU-seq) from progeria cohort:<br>EU-seq Ercc1Δ/- 4 weeks 1<br>EU-seq Ercc1Δ/- 4 weeks 2<br>EU-seq Ercc1Δ/- 4 weeks 3<br>EU-seq Ercc1Δ/- 10 weeks 1<br>EU-seq Ercc1Δ/- 10 weeks 2<br>EU-seq Ercc1Δ/- 10 weeks 3<br>EU-seq Xpg-/- 7 weeks 1<br>EU-seq Xpg-/- 7 weeks 2<br>EU-seq Xpg-/- 7 weeks 3<br>EU-seq Xpg-/- 14 weeks 1<br>EU-seq Xpg-/- 14 weeks 2<br>EU-seq Xpg-/- 14 weeks 3<br>EU-seq WT progeria cohort 4wk 1<br>EU-seq WT progeria cohort 4wk 2<br>EU-seq WT progeria cohort 4wk 3<br>EU-seq WT progeria cohort 14wk 1<br>EU-seq WT progeria cohort 14wk 2<br>EU-seq WT progeria cohort 14wk 3<br><br>Nascent RNA-seq (EU-seq) from normally aged cohort:<br>EU-seq WT adult liver 1<br>EU-seq WT adult liver 2<br>EU-seq WT adult liver 3<br>EU-seq WT old liver 1<br>EU-seq WT old liver 2<br>EU-seq WT old liver 3<br>EU-seq WT adult kidney 1<br>EU-seq WT adult kidney 2<br>EU-seq WT old kidney 1<br>EU-seq WT old kidney 2
Genome browser session<br>(e.g. UCSC)	n.a.

Methodology

Replicates	Nascent RNA-seq and ChIP-seq was done in triplicates.
Sequencing depth	64-119M reads
Antibodies	RNAPII-ser2p (Abcam, ab5095)<br>RNAPII-ser5p (Abcam, ab5131)<br>RNAPII RPB1-NTD (Cell signaling, D8L4Y)
Peak calling parameters	Expressed genes were analyzed with annotated locations, thus no new peak calling was needed.
Data quality	Percentage of read position with a base quality score above 30 ranges between 90.32% - 93-31%
Software	Reads were mapped to Mus musculus genome version 10 (mm10) using Bowtie.<br>Read quality was assessed using FastQC and FastQScreen.<br>Annotated expressed genes were binned by K_bining.py.<br>Read coverage on annotated genes was quantified by HTseq.<br>Coverage profiles on genes was plotted by using HOMER software.
